# Supplementary material for: Screening and diversity of culturable HNAD bacteria in the MBR sewage treatment system
Source: PLoS One. 2024 Jan 18;19(1):e0293136. doi: 10.1371/journal.pone.0293136 (PMC10796061; doi:10.1371/journal.pone.0293136)
Supplement: Attachment — Submitted filename: Response to Reviewers.docx [file pone.0293136.s001.docx]

**Response to Reviewers**

Reviewer #1:

1. L44. The traditional nitrification process utilized autotrophic nitrifiers rather than heterotrophic nitrifiers.

Reply: Accepted and it was corrected.

2. L111-112. How was the bacterial suspension with suitable dilution gradients cultivated?

Reply: Accepted and it was corrected. Bacterial suspensions do not need to be cultured and can be directly streaked.

3. L145-147. Alkaline potassium persulfate digestion is used to measure total nitrogen. How are nitrate nitrogen and ammonia nitrogen measured in this manuscript?

Reply:Accepted and it has been added.

4. L163. There are 53 HNAD strains reported in the manuscript, but only 26 in Table 1. What are the differences and novelties between the isolated HNAD bacteria and those reported?

Reply:Accepted and it was improved.

Reviewer #2:

1. Abstract: Heterophic nitrifying ability of isolateds HAND strains shuold be also tested and presented.

Reply:Accepted and it was improved.

2. Table 1 could be deleted

Reply: Accepted and it has been deleted.

3. Tables 2 & 3: Growth (OD600) of the strains under these conditions needs to be also presented.

Reply:Accepted and it has been added.

4. L 28: (bacterial strain)

Reply: Accepted and it was corrected.

5. L 44: Heterotrophic nitrifiers?

Reply: Accepted and it was corrected.

6. L 85: LB broth

Reply: Accepted and it was corrected.

7. L 87: Enrichment medium

Reply: Accepted and it was corrected.
